# P-1939. Characteristics, Treatment, and Outcomes of Fluconazole-Susceptible and Non-Susceptible Candidemia: A Large Network Analysis

**DOI:** 10.1093/ofid/ofaf695.2107

**Published:** 2026-01-11

**Authors:** Isabel C Campa, Molly C Studebaker, Martin Krsak, Andrés F Henao Martínez,, Daniel B Chastain

**Affiliations:** University of Georgia College of Pharmacy, Valdosta, GA; University of Georgia College of Pharmacy, Valdosta, GA; University of Colorado School of Medicine, CO; University of Colorado Anschutz Medical Campus, Aurora, Colorado; University of Georgia College of Pharmacy, Valdosta, GA

## Abstract

**Background:**

The increasing prevalence of fluconazole non-susceptible (FNS) *Candida* species and limited access to susceptibility testing emphasize the need to identify factors associated with these infections. Understanding the impact of fluconazole susceptibility on clinical outcomes is also crucial. This study compared the characteristics, treatments, and outcomes of fluconazole-susceptible (FS) versus FNS candidemia.

**Figure:**
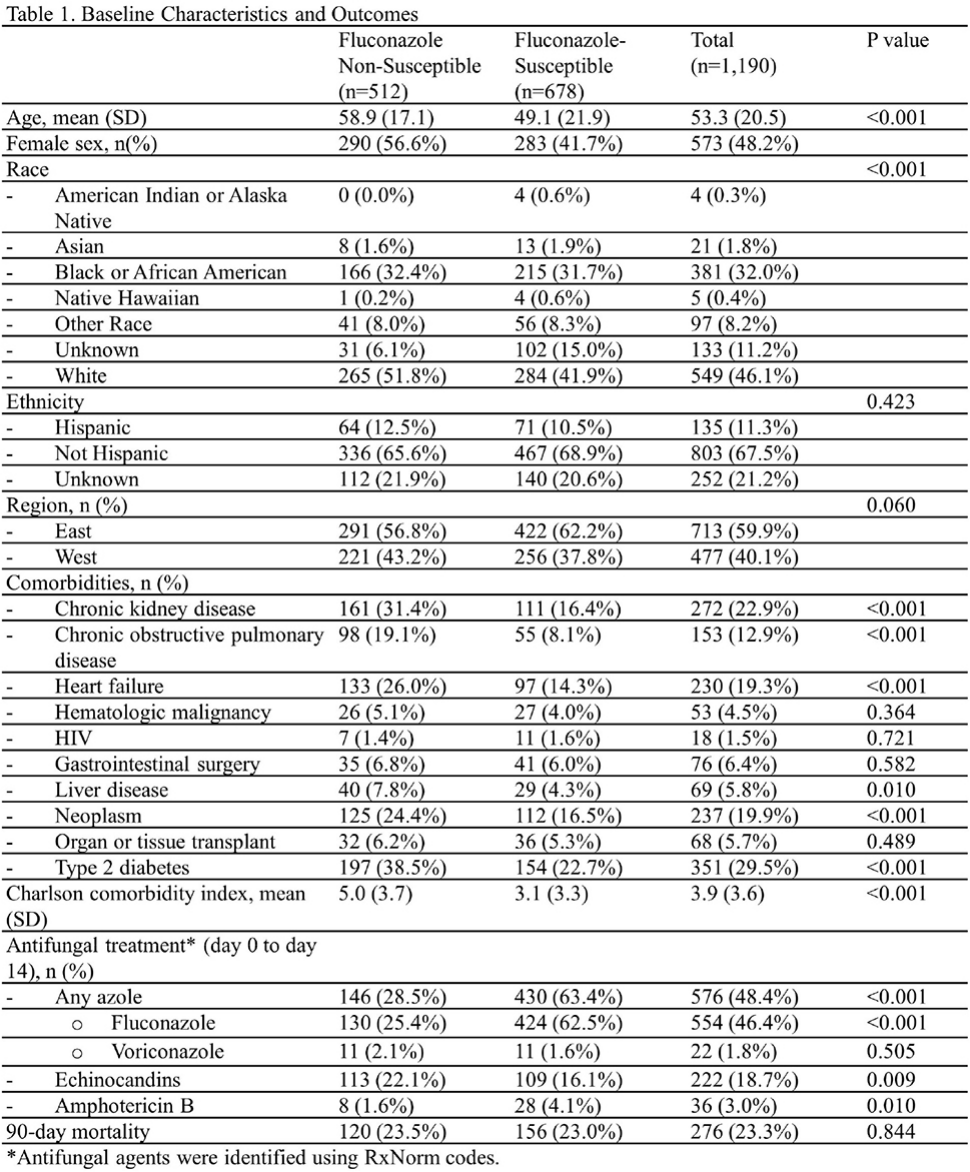

**Figure:**
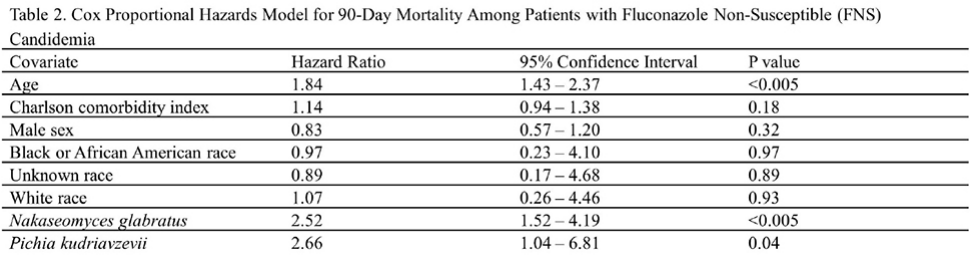

**Methods:**

Adults (≥18 years) with candidemia, defined by positive PCR blood tests, were identified within the TriNetX federated network (May 2017–November 2023). Cases were classified based on presumed fluconazole susceptibility: species typically FS (*C. albicans*, *C. parapsilosis*, *C. tropicalis*) versus typically FNS (*Nakaseomyces glabratus*, *Pichia kudriavzevii*, *C. auris*). Propensity scores, based on demographics and comorbidities, were used to generate Inverse Probability of Treatment Weights (IPTW). Weighted Cox proportional hazards models assessed outcomes, and IPTW-adjusted Kaplan-Meier curves with Monte Carlo bootstrapping evaluated survival.

**Figure:**
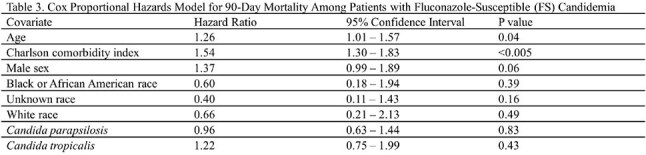

**Figure:**
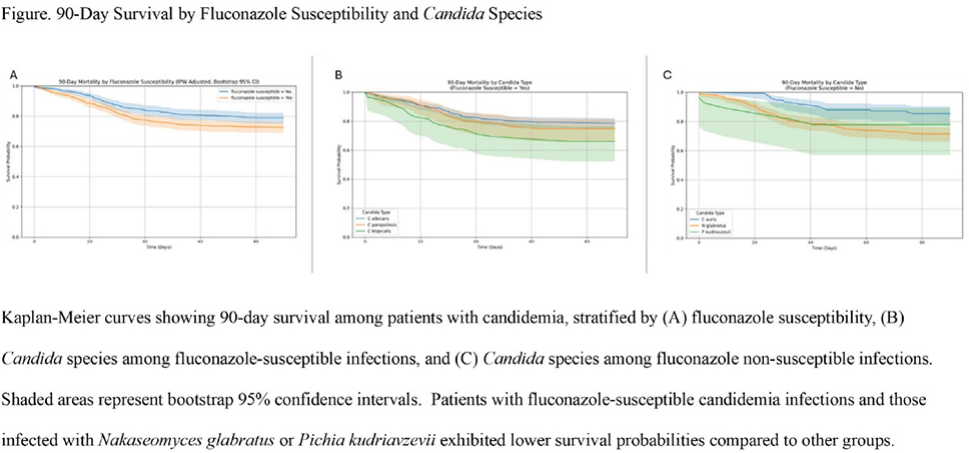

**Results:**

Of 1190 candidemia cases, 43% were FNS. Patients with FNS candidemia were older, more likely female, with a higher comorbidity burden (Table 1). FS cases were more frequently treated with fluconazole (63% vs 25%, p< .001), whereas FNS cases more often received an echinocandin (22% vs 16%, p=.009) or amphotericin B (p=.010). In the Cox model for FNS candidemia, older age (HR 1.84, p< .005), *N. glabratus* (HR 2.52, p< .005), and *P. kudriavzevii* (HR 2.66, p=.04) were associated with higher mortality (Table 2). For FS candidemia, older age (HR 1.26, p=.04) and higher Charlson comorbidity index (HR 1.54, p< .005) were associated with increased mortality risk (Table 3). Kaplan-Meier survival analysis showed that patients with FS candidemia and those with *N. glabratus* or *P. kudriavzevii* had lower 90-day survival probabilities compared to other groups (Fig).

**Conclusion:**

Nearly half of candidemia cases involved FNS species. Notably, older age and higher comorbidity independently increased mortality in FS candidemia, which was associated with lower survival compared to FNS candidemia. This unexpected finding may reflect illness severity, suboptimal initial therapy, or host factor differences in FS candidemia.

**Disclosures:**

Andrés F. Henao Martínez, MD, MPH, F2: Grant/Research Support|Scynexis: Grant/Research Support

